# Novel Hydrogen Sulfide (H_2_S)-Releasing BW-HS-101 and Its Non-H_2_S Releasing Derivative in Modulation of Microscopic and Molecular Parameters of Gastric Mucosal Barrier

**DOI:** 10.3390/ijms22105211

**Published:** 2021-05-14

**Authors:** Dominik Bakalarz, Edyta Korbut, Zhengnan Yuan, Bingchen Yu, Dagmara Wójcik, Aleksandra Danielak, Katarzyna Magierowska, Slawomir Kwiecień, Tomasz Brzozowski, Monika Marcinkowska, Binghe Wang, Marcin Magierowski

**Affiliations:** 1 Department of Physiology, Jagiellonian University Medical College, 31531 Cracow, Poland; dominik.bakalarz@uj.edu.pl (D.B.); edyta.korbut@uj.edu.pl (E.K.); dagmara1.wojcik@uj.edu.pl (D.W.); aleksandra26.danielak@student.uj.edu.pl (A.D.); katarzyna.magierowska@uj.edu.pl (K.M.); skwiecien@cm-uj.krakow.pl (S.K.); mpbrzozo@cyf-kr.edu.pl (T.B.); 2 Department of Forensic Toxicology, Institute of Forensic Research, 31033 Cracow, Poland; 3 Department of Chemistry and Center for Diagnostics and Therapeutics, Georgia State University, Atlanta, GA 30302, USA; zyuan3@gsu.edu (Z.Y.); yubingchen2013@gmail.com (B.Y.); 4 Faculty of Pharmacy, Jagiellonian University Medical College, 30688 Cracow, Poland; monika.marcinkowska@uj.edu.pl

**Keywords:** hydrogen sulfide prodrugs, BW-HS-101, non-steroidal anti-inflammatory drugs, gastrotoxicity, molecular gastroenterology, gastrointestinal pharmacology

## Abstract

Hydrogen sulfide (H_2_S) is an endogenously produced molecule with anti-inflammatory and cytoprotective properties. We aimed to investigate for the first time if a novel, esterase-sensitive H_2_S-prodrug, BW-HS-101 with the ability to release H_2_S in a controllable manner, prevents gastric mucosa against acetylsalicylic acid-induced gastropathy on microscopic and molecular levels. Wistar rats were pretreated intragastrically with vehicle, BW-HS-101 (0.5–50 μmol/kg) or its analogue without the ability to release H_2_S, BW-iHS-101 prior to ASA administration (125 mg/kg, intragastrically). BW-HS-101 was administered alone or in combination with nitroarginine (L-NNA, 20 mg/kg, intraperitoneally) or zinc protoporphyrin IX (10 mg/kg, intraperitoneally). Gastroprotective effects of BW-HS-101 were additionally evaluated against necrotic damage induced by intragastrical administration of 75% ethanol. Gastric mucosal damage was assessed microscopically, and gastric blood flow was determined by laser flowmetry. Gastric mucosal DNA oxidation and PGE_2_ concentration were assessed by ELISA. Serum and/or gastric protein concentrations of IL-1α, IL-1β, IL-2, IL-4, IL-6, IL-10, IL-13, VEGF, GM-CSF, IFN-γ, TNF-α, and EGF were determined by a microbeads/fluorescent-based multiplex assay. Changes in gastric mucosal iNOS, HMOX-1, SOCS3, IL1-R1, IL1-R2, TNF-R2, COX-1, and COX-2 mRNA were assessed by real-time PCR. BW-HS-101 or BW-iHS-101 applied at a dose of 50 μmol/kg protected gastric mucosa against ASA-induced gastric damage and prevented a decrease in the gastric blood flow level. H_2_S prodrug decreased DNA oxidation, systemic and gastric mucosal inflammation with accompanied upregulation of SOCS3, and EGF and HMOX-1 expression. Pharmacological inhibition of nitric oxide (NO) synthase but not carbon monoxide (CO)/heme oxygenase (HMOX) activity by L-NNA or ZnPP, respectively, reversed the gastroprotective effect of BW-HS-101. BW-HS-101 also protected against ethanol-induced gastric injury formation. We conclude that BW-HS-101, due to its ability to release H_2_S in a controllable manner, prevents gastric mucosa against drugs-induced gastropathy, inflammation and DNA oxidation, and upregulate gastric microcirculation. Gastroprotective effects of this H_2_S prodrug involves endogenous NO but not CO activity and could be mediated by cytoprotective and anti-inflammatory SOCS3 and EGF pathways.

## 1. Introduction

Non-steroidal anti-inflammatory drugs (NSAIDs), including acetylsalicylic acid (ASA, the active ingredient of aspirin), exert analgesic, anti-pyretic, and anti-inflammatory effects. The mechanism of action of ASA is through the inhibition of cyclooxygenases (COX-1, COX-2), enzymes involved in the synthesis of pro-inflammatory but also cytoprotective prostaglandins (PGs) such as PGE_2_. However, inhibition of COXs also reduces thromboxane synthesis, leading to decreased platelet aggregation and an antithrombotic effect. Therefore, ASA is widely used for prevention of ischemic stroke and myocardial infarction [1]. Despite the beneficial effects of ASA on the cardiovascular system, the adverse effects of this drug within the gastrointestinal (GI) tract, such as gastric mucosal injuries, remain a major limitation of its therapeutic effectiveness in humans [2]. The pathogenesis of this ASA-induced gastrotoxicity is attributed to its topical and systemic effects [3,4] since this drug irreversibly inhibits COX-1 and COX-2. Deficiency of PGs in the gastric mucosa is involved in the impairment of gastric mucosal integrity. Moreover, ASA itself is chemically irritating and cytotoxic for gastric epithelial cells [5].

Hydrogen sulfide (H_2_S) is an intracellular gaseous signaling molecule and participates in many physiological and pathological processes within the GI tract and in other parts of the body [6,7]. Numerous studies have shown that H_2_S serves as an anti-oxidative, anti-inflammatory, and cytoprotective agent [8,9]. For instance, H_2_S has been reported to inhibit the adherence of leukocytes to blood vessel walls [10]. This simple inorganic molecule has also been shown to have a vasodilatory effect, similar to its sister endogenous gaseous mediators, carbon monoxide (CO) and nitric oxide (NO) [11,12,13,14,15]. H_2_S is produced endogenously by the metabolism of L-cysteine, mainly with the involvement of two pyridoxal-5-phosphate (P5P, vitamin B6)-dependent enzymes: cystathionine-γ-lyase (CTH) and cystathionine-β-synthase (CBS) [16,17]. Alternatively, H_2_S is biosynthesized with the participation of mercaptopyruvate-3-sulfurtransferase (MPST) with the coactivity of cysteine aminotransferase [18]. H_2_S can also be produced from sulfates without enzymatic activity by colonic bacteria [19].

Previous studies have shown that diallyl disulfide (DADS), a H_2_S donor derived from garlic, inhibited proliferation of human colon cancer HT29 cells in a dose-dependent manner [20]. Moreover, pretreatment with sodium hydrosulfide (NaHS) prevented NSAIDs-induced gastrotoxicity [21]. It has been shown that H_2_S inhibited lipid peroxidation and formation of acute stress-induced or ischemia/reperfusion-gastric damage [22,23]. Wallace et al. observed that daily intragastric treatment with L-cysteine or Lawesson’s reagent, a H_2_S donor, accelerated the healing of experimental chronic gastric ulcers [24]. In addition to its gastroprotective effects, NaHS was also observed to increase dose-dependently the secretion of HCO_3_^−^ ions in the duodenum of rats, which resulted in the maintenance of intestinal mucosal integrity [25]. Interestingly, pretreatment with a synthetic slowly-release H_2_S donor, GYY4137, reduced ischemia/reperfusion-induced gastric injury in rats [22]. Importantly, GI-safe H_2_S-releasing derivatives of conventional NSAIDs have been developed recently. ATB-346, a naproxen derivative conjugated with 4-hydroxythiobenzamide, has been shown to have significantly reduced gastrotoxicity in pre-clinical experiments and in phase-2 clinical trials [26]. Therefore, the implementation of novel H_2_S-releasing molecules seems to be a rational approach for the development of treatment options for pathologies in the GI. BW-HS-101 is a novel organic and esterase sensitive H_2_S-releasing compound, which in contrast to sulfide salts like NaHS exerts controlled release rates of this gaseous mediator with a half-life about 13 min [27]. This H_2_S prodrug has been reported to decrease lipopolysaccharide (LPS)-induced inflammatory responses in vitro [27].

**Figure 1:**
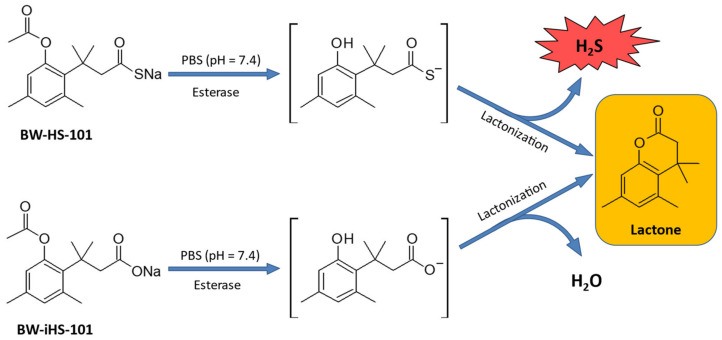
Hydrolysis of BW-HS-101 and BW-iHS-101 leading to the generation of lactone and hydrogen sulfide (H_2_S) or water (H_2_O), respectively.

In this study, we aimed to investigate for the first time if pretreatment with a novel H_2_S prodrug, BW-HS-101 applied intragastrically (i.g.), protects gastric mucosa against ASA-induced injury and if this compound affects gastric blood flow (GBF). We have included the analogue of the H_2_S-prodrug, BW-iHS-101, without the ability to release this gaseous mediator in this study (Figure 1). We aimed to additionally confirm the possible gastroprotective effect of BW-HS-101 against necrotic injuries induced by i.g. administration of 75% ethanol. We focused on possible modulation of systemic and GI inflammatory responses and alterations in molecular patterns. We also examined the possible involvement of endogenous CO and NO produced by heme oxygenase (HMOX) or NO synthase (NOS) activities, respectively, in gastric mucosal integrity maintenance by BW-HS-101.

## 2. Results

### 2.1. Chemical Conversion of BW-HS-101 and BW-iHS-101

Figure 1 demonstrates the biochemical conversions of BW-HS-101 and BW-iHS-101. Due to the activity of esterase, both compounds are hydrolyzed leading to the generation of the lactone product as an inactive metabolite, and H_2_S or H_2_O for BW-HS-101 and BW-iHS-101, respectively (Figure 1).

### 2.2. Possible Effects on the Gastric Damage Score, Gastric Blood Flow Alterations, and Systemic and Gastric Mucosal Inflammation. Pharmacological Evaluation of the Involvement of Endogenous NO and CO Biosynthesis Pathways

**Figure 2:**
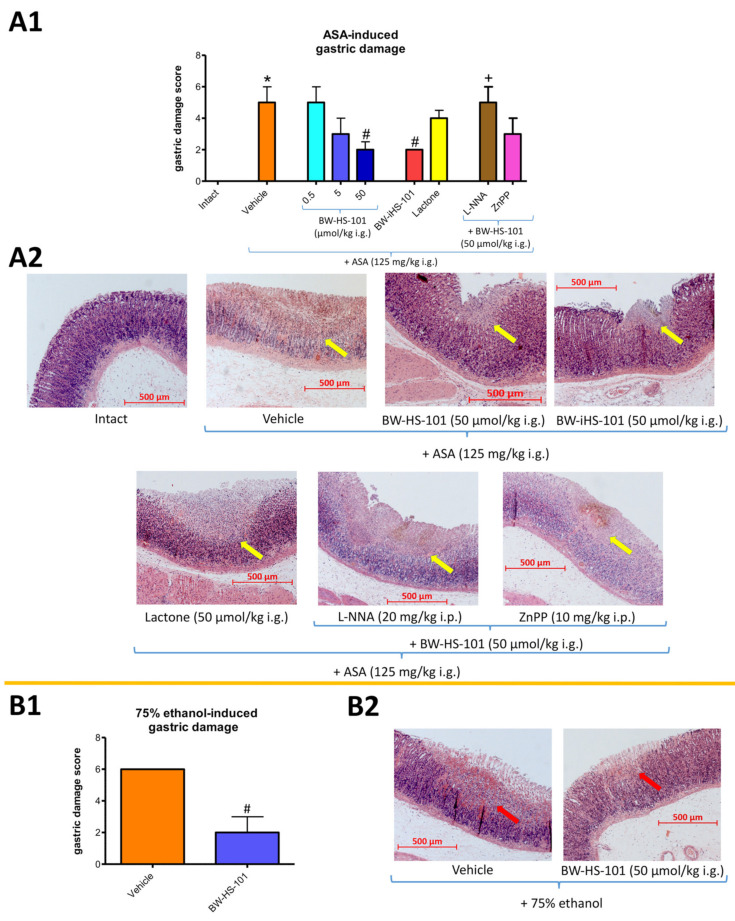
Gastric damage score (**A1**,**B1**) and representative histological slides (**A2**,**B2**) of gastric mucosa of rats administered i.g. with acetylsalicylic acid (ASA, 125 mg/kg) or 1 mL of 75% ethanol. Results are median ± interquartile range of five rats per each experimental group. Asterisk (*) indicates significant change as compared with intact (*p* < 0.05). Hash (#) indicates significant change as compared with vehicle (*p* < 0.05). Cross (+) indicates significant change as compared with BW-HS-101 (50 μmol/kg) (*p* < 0.05). (**A1**): Gastric damage score in rats administered with ASA and pretreated i.g. 30 min earlier with vehicle, BW-HS-101 (0.5–50 μmol/kg), BW-iHS-101 (50 μmol/kg), lactone (50 μmol/kg) or BW-HS-101 (50 μmol/kg) combined with nitroarginine (L-NNA, 20 mg/kg i.p.) or zinc protoporphyrin IX (ZnPP, 10 mg/kg i.p.). (**A2**): Representative histological slides of gastric mucosal damage induced by ASA (yellow arrows) in rats pretreated i.g. with vehicle, BW-HS-101 (50 μmol/kg), BW-iHS-101 (50 μmol/kg), lactone (50 μmol/kg), or BW-HS-101 (50 μmol/kg) combined with L-NNA or ZnPP. (**B1**): Gastric damage score in rats administered with 75% ethanol and pretreated i.g. 30 min earlier with vehicle or BW-HS-101 (50 μmol/kg). (**B2**): Representative histological slides of gastric mucosal damage induced by 75% ethanol (red arrows) in rats pretreated i.g. with vehicle or BW-HS-101 (50 μmol/kg).

Figure 2(A1) shows that pretreatment with BW-HS-101 applied i.g. at a dose of 50 but not 0.5 or 5 μmol/kg significantly decreased ASA-induced gastric damage score by more than 50% as compared with vehicle-treated rats (*p* < 0.05). BW-iHS-101 (50 μmol/kg i.g.) but not lactone (50 μmol/kg i.g.) significantly decreased the ASA-induced gastric damage score as compared with vehicle (Figure 2(A1); *p* < 0.05). Additionally, Figure 2(A1) shows that i.g. pretreatment with nitroarginine (L-NNA, 20 mg/kg applied intraperitoneally (i.p.)) but not zinc protoporphyrin IX (ZnPP, 10 mg/kg i.p.) in combination with BW-HS-101 (50 μmol/kg i.g.) significantly decreased ASA-induced gastric damage score as compared with rats administered with BW-HS-101 (50 μmol/kg i.g.) alone (*p* < 0.05).

Figure 2(A2) shows representative microscopic photomicrographs of gastric mucosa pretreated with vehicle, BW-HS-101, BW-iHS-101 or lactone applied i.g. at a dose of 50 μmol/kg and administered with ASA (125 mg/kg i.g.). Topical application of ASA induced hemorrhagic erosions of the epithelial surface penetrating more than 250 μm into gastric mucosa, even reaching lamina propria with the width being more than 500 μm and with submucosal leukocytes infiltration (Figure 2(A2)). These alterations were not observed in intact gastric mucosa (Figure 2(A2)). Pretreatment with BW-HS-101 or BW-iHS-101 but not with lactone limited bleeding and the injury range to the superficial epithelium (Figure 2(A2)). Pretreatment with BW-HS-101 applied in combination with ZnPP but not with L-NNA limited gastric damage to the superficial epithelium (Figure 2(A2)).

Figure 2(B1) shows that pretreatment with BW-HS-101 (50 μmol/kg i.g.) significantly decreased ethanol-induced gastric damage score as compared with vehicle-treated rats (*p* < 0.05). Figure 2(B2) shows that topical application of 75% ethanol induced necrotic and hemorrhagic erosions of the epithelial surface penetrating more than 250 μm into the lamina propria of gastric mucosa with the width being more than 500 μm and with submucosal leukocytes infiltration. Pretreatment with BW-HS-101 reduced bleeding and limited the gastric erosion development to the superficial epithelium (Figure 2(B2)).

Administration of ASA significantly decreased the GBF as compared with intact gastric mucosa (*p* < 0.05) (Table 1). Pretreatment with BW-HS-101 (50 μmol/kg i.g.) or BW-iHS-101 (50 μmol/kg i.g.) but not lactone (50 μmol/kg i.g.) significantly increased GBF in rats with ASA-induced gastric damage as compared to the group pretreated with vehicle (*p* < 0.05, Table 1). Pretreatment with BW-HS-101 (50 μmol/kg i.g.) significantly increased GBF as compared with BW-iHS-101 (50 μmol/kg i.g.) (*p* < 0.05, Table 1). Pretreatment with L-NNA (20 mg/kg i.p.) but not with ZnPP (10 mg/kg i.p.) in combination with BW-HS-101 (50 μmol/kg i.g.) significantly decreased GBF in rats with ASA-induced gastric damage as compared to the group pretreated with BW-HS-101 applied alone (*p* < 0.05, Table 1). Pretreatment with ZnPP (10 mg/kg i.p.) but not with L-NNA (20 mg/kg i.p.) in combination with BW-HS-101 (50 μmol/kg i.g.) significantly increased GBF in rats with ASA-induced gastric damage as compared to the group pretreated with vehicle (*p* < 0.05, Table 1).

**Figure 3:**
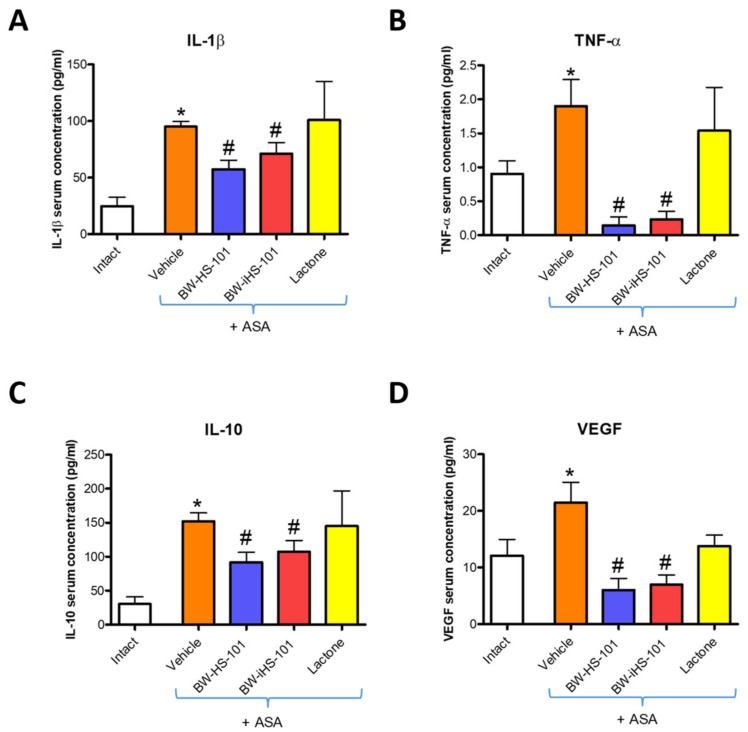
Concentration of interleukin (IL)-1β (**A**), tumor necrosis factor (TNF)-α (**B**), IL-10 (**C**), or vascular endothelial growth factor A (VEGF) (**D**) in serum of rats administered i.g. with acetylsalicylic acid (ASA, 125 mg/kg) and pretreated 30 min earlier with vehicle, BW-HS-101 (50 μmol/kg), BW-iHS-101 (50 μmol/kg), or lactone (50 μmol/kg). Results are mean ± SEM of five rats per each experimental group. Significant changes as compared with the respective values in intact rats are indicated by asterisk (*) (*p* < 0.05). Hash (#) indicates significant changes as compared with vehicle (*p* < 0.05).

Figure 3 shows that in rats administered with ASA, serum concentrations of IL-1β (A), TNF-α (B), IL-10 (C), and VEGFA (D) were significantly increased as compared with intact animals (*p* < 0.05). Pretreatment with BW-HS-101 (50 μmol/kg) and BW-iHS-101 (50 μmol/kg) but not with lactone (50 μmol/kg) significantly decreased serum concentrations of IL-1β (A), TNF-α (B), IL-10 (C) and VEGFA (D) as compared with vehicle (*p* < 0.05).

**Figure 4:**
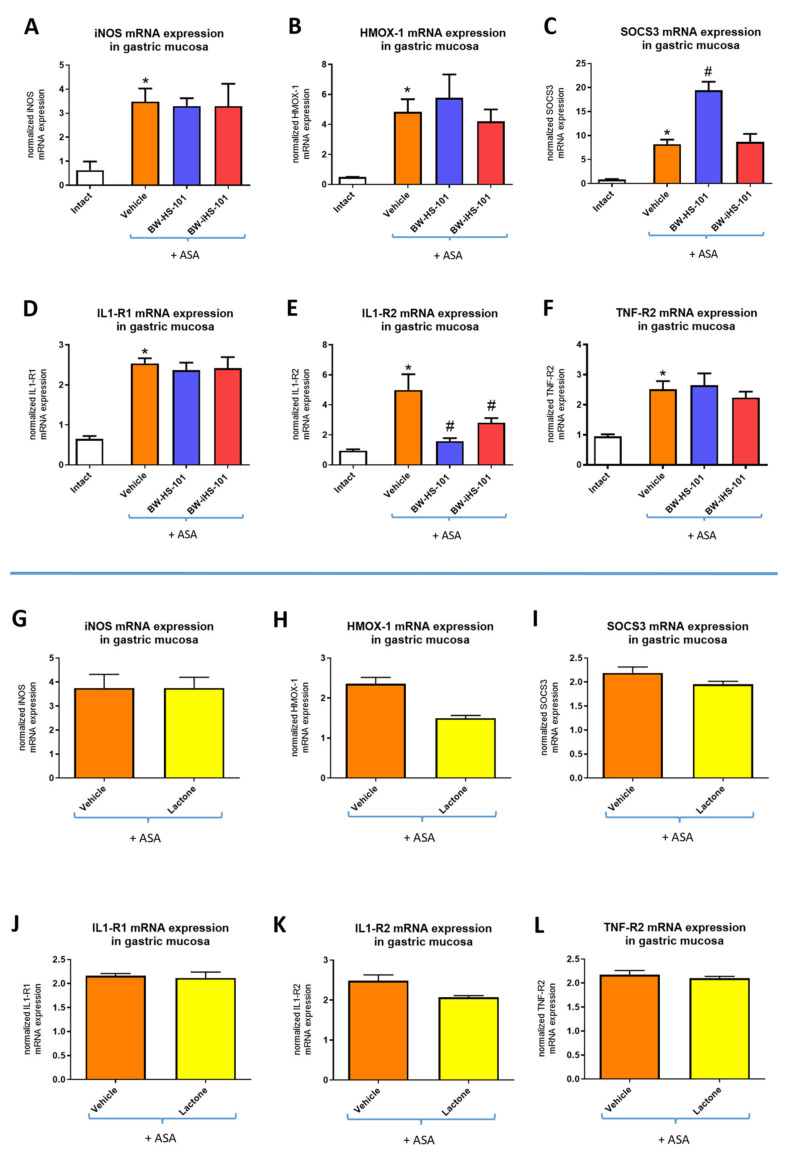
Gastric mucosal mRNA expression of inducible nitric oxide synthase (iNOS) (**A**,**G**), heme oxygenase (HMOX)-1 (**B**,**H**), suppressor of cytokine signaling 3 (SOCS3) (**C**,**I**), interleukin (IL) receptor (R)1 (**D**,**J**), IL-R2 (**E**,**K**), and tumor necrosis factor (TNF)-R2 (**F**,**L**) in rats administered i.g. with acetylsalicylic acid (ASA, 125 mg/kg) and pretreated 30 min earlier with vehicle, BW-HS-101 (50 μmol/kg), BW-iHS-101 (50 μmol/kg) or lactone (50 μmol/kg). Results are mean ± SEM of five rats per each experimental group. Significant changes as compared with the respective values in intact rats are indicated by asterisk (*) (*p* < 0.05). Hash (#) indicates significant changes as compared with vehicle (*p* < 0.05). (**G**–**L**): Results were reported on separate figures because technically the data were calculated based on the different reference sample and the fold-change values for the same vehicle group are different than on panels (**A**–**F**).

Figure 4A–F shows that in rats administered with ASA, gastric mucosal mRNA expression of inducible NOS (iNOS) (A), HMOX-1 (B), suppressor of cytokine signaling 3 (SOCS3) (C), interleukin (IL)1-receptor 1 (R1) (D), IL-1R2 (E), tumor necrosis factor (TNF)-receptor 2 (R2) (F) was significantly increased as compared with intact animals (*p* < 0.05). Pretreatment with BW-HS-101 (50 μmol/kg) or BW-iHS-101 (50 μmol/kg) significantly decreased IL-1R2 mRNA expression (E) but did not significantly affected mRNA expression of iNOS (A), HMOX-1 (B), IL1-R1 (D), and TNF-R2 (F) as compared with vehicle (Figure 4). BW-HS-101 but not BW-iHS-101 significantly upregulated SOCS3 mRNA expression as compared with vehicle (*p* < 0.05, Figure 4C). Figure 4G–L shows that in rats pretreated with lactone (50 μmol/kg), gastric mucosal mRNA expression of iNOS (G), HMOX-1 (H), SOCS3 (I), IL1-R1 (J), IL-1R2 (K), TNF-R2 (L) was not significantly altered as compared with vehicle.

### 2.3. Molecular Pattern of Gastric Mucosal Proteins

**Figure 5:**
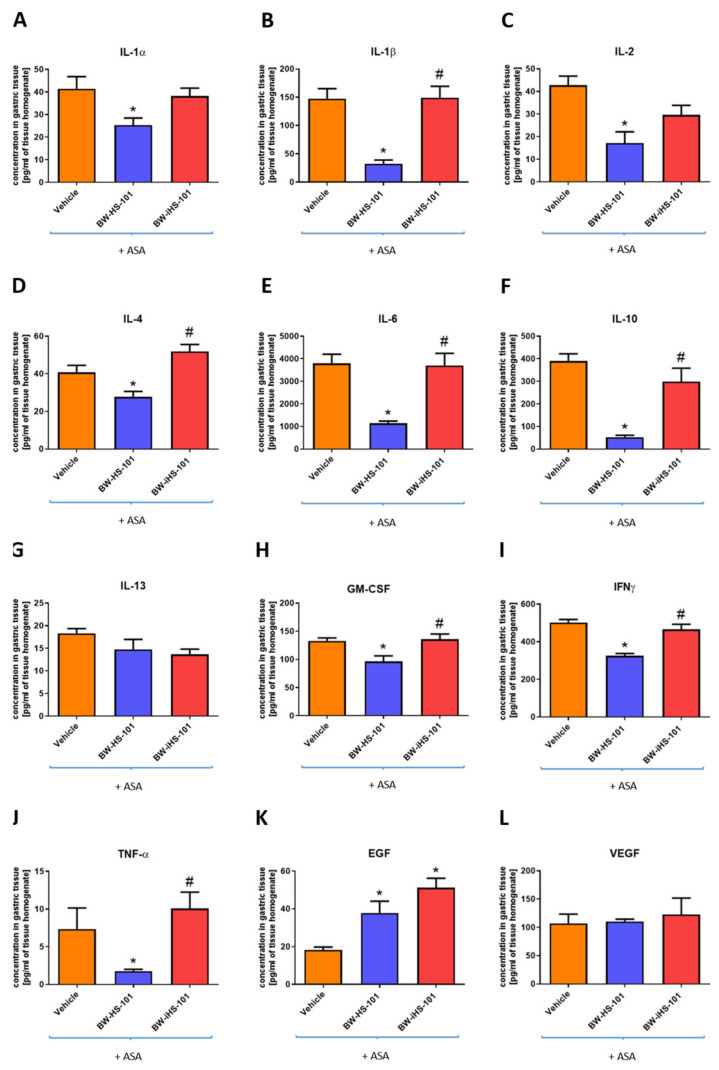
Concentration of interleukin (IL)-1α (**A**), IL-1β (**B**), IL-2 (**C**), IL-4 (**D**), IL-6 (**E**), IL-10 (**F**), IL-13 (**G**), granulocyte-macrophage colony-stimulating factor (GM-CSF) (**H**), interferon (IFN) γ (**I**), tumor necrosis factor (TNF)-α (**J**), epidermal growth factor (EGF) (**K**), or vascular endothelial growth factor (VEGF) (**L**) in gastric mucosa of rats administered i.g. with acetylsalicylic acid (ASA, 125 mg/kg) and pretreated 30 min earlier with vehicle, BW-HS-101 (50 μmol/kg), BW-iHS-101 (50 μmol/kg) or lactone (50 μmol/kg). Results are mean ± SEM of five rats per each experimental group. Significant changes as compared with the respective values in vehicle-treated rats are indicated by asterisk (*) (*p* < 0.05). Hash (#) indicates significant changes as compared with BW-HS-101 (*p* < 0.05).

Figure 5 shows that pretreatment with BW-HS-101 (50 μmol/kg i.g.) but not with BW-iHS-101 (50 μmol/kg i.g.) significantly decreased gastric mucosal concentration of IL-1α (A), IL-1β (B), IL-2 (C), IL-4 (D), IL-6 (E), IL-10 (F), granulocyte-macrophage colony-stimulating factor (GM-CSF) (H), interferon (IFN)γ (I), TNF-α (J) as compared with vehicle in rats administered with ASA (*p* < 0.05). Figure 5 shows that BW-HS-101 and BW-iHS-101 significantly increased gastric mucosal protein concentration of epidermal growth factor (EGF) (K) but not vascular endothelial growth factor A (VEGF) (L) or IL-13 (G) as compared with vehicle in rats administered with ASA (*p* < 0.05). Based on the above-reported data, the lactone-pretreated group was not included in this experiment. Alterations in inflammatory and oxidative response markers within gastric mucosa exposed to ASA vs. intact were also in part confirmed as shown on Figure 3 and Figure 4. The intact group was also not analyzed within this experimental series, since the alterations in the abovementioned targets concentration on a systemic level after administration of ASA vs. intact were previously reported [28].

### 2.4. Possible Alterations in the Gastric Mucosal Prostaglandins/Cyclooxygenase Pathway Activity

**Figure 6:**
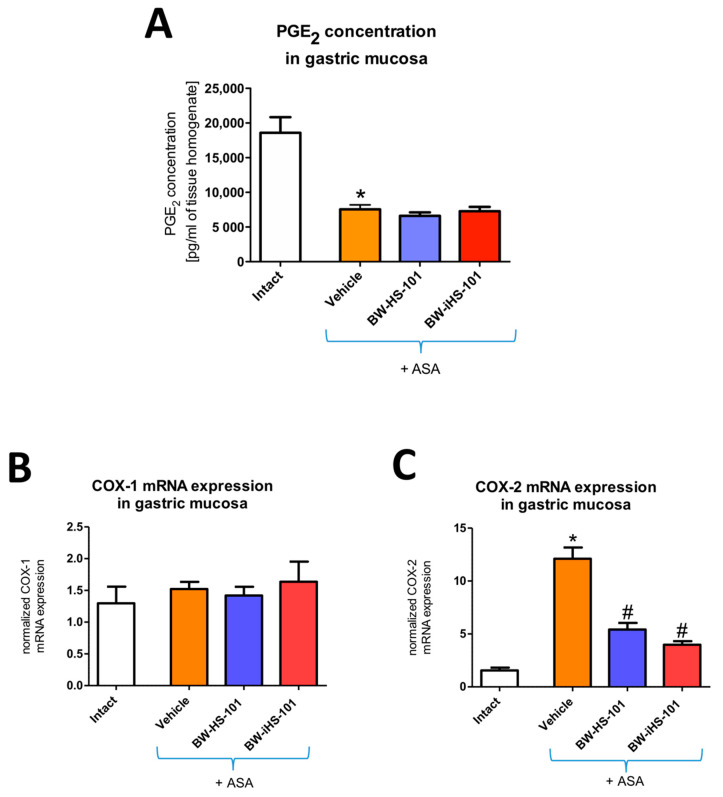
Gastric mucosal concentration of prostaglandin E_2_ (**A**) and mRNA expression of cyclooxygenase (COX)-1 (**B**) and COX-2 (**C**) in rats administered i.g. with acetylsalicylic acid (ASA, 125 mg/kg) and pretreated 30 min earlier with vehicle, BW-HS-101 (50 μmol/kg) or BW-iHS-101 (50 μmol/kg). Results are mean ± SEM of five rats per each experimental group. Significant changes as compared with the respective values in intact rats are indicated by asterisk (*) (*p* < 0.05). Hash (#) indicates significant changes as compared with vehicle (*p* < 0.05).

Figure 6A shows that in rats administered with ASA gastric mucosal concentration of PGE_2_ was significantly decreased as compared with intact animals (*p* < 0.05). Pretreatment with BW-HS-101 (50 μmol/kg) or BW-iHS-101 (50 μmol/kg) did not significantly affected PGE_2_ concentration as compared with vehicle (Figure 6A). Figure 6B shows that in rats administered with ASA gastric mucosal mRNA expression of COX-1 was not significantly altered as compared with intact animals. Pretreatment with BW-HS-101 (50 μmol/kg) or BW-iHS-101 (50 μmol/kg) did not significantly affected COX-1 mRNA expression as compared with vehicle (Figure 6B). Figure 6C shows that in rats administered with ASA gastric mucosal mRNA expression of COX-2 was significantly upregulated as compared with intact animals (*p* < 0.05). Pretreatment with BW-HS-101 (50 μmol/kg) or BW-iHS-101 (50 μmol/kg) significantly decreased COX-2 mRNA expression as compared with vehicle (*p* < 0.05, Figure 6C). Based on the above-reported data, the lactone-pretreated group was not included in this experiment.

### 2.5. Oxidation of DNA in Gastric Mucosa

**Figure 7:**
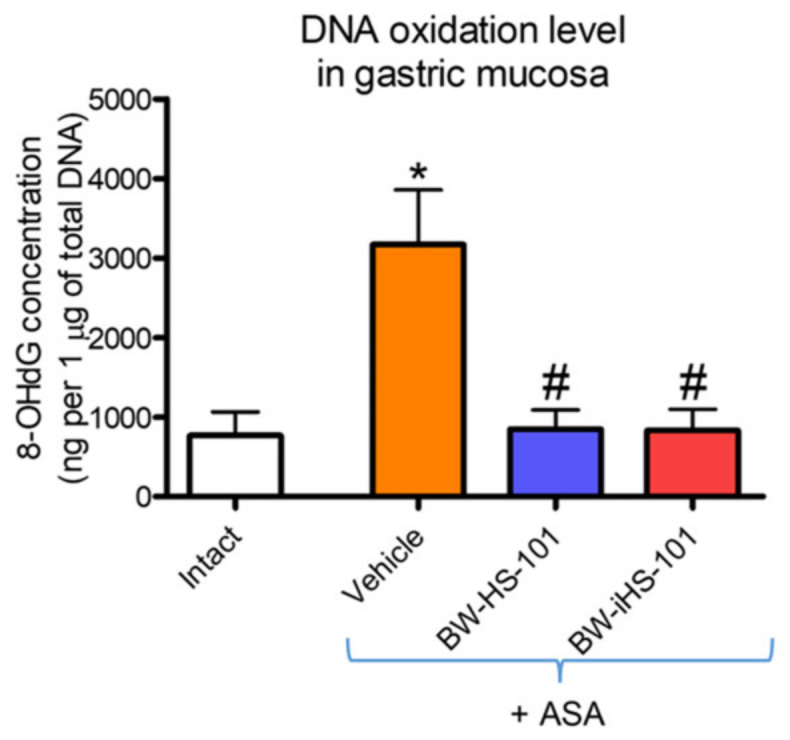
Gastric mucosal concentration of 8-hydroxy-deoxyguanosine (8-OHdG) in rats administered i.g. with acetylsalicylic acid (ASA, 125 mg/kg) and pretreated 30 min earlier with vehicle, BW-HS-101 (50 μmol/kg) or BW-iHS-101 (50 μmol/kg). Results are mean ± SEM of five rats per each experimental group. Significant changes as compared with the respective values in intact rats are indicated by asterisk (*) (*p* < 0.05). Hash (#) indicates significant changes as compared with vehicle (*p* < 0.05).

Figure 7 shows that in rats administered with ASA gastric mucosal concentration of 8-hydroxy-deoxyguanosine (8-OHdG) was significantly increased as compared with intact animals (*p* < 0.05). Pretreatment with BW-HS-101 (50 μmol/kg) or BW-iHS-101 (50 μmol/kg) significantly decreased gastric mucosal 8-OHdG concentration as compared with vehicle (*p* < 0.05, Figure 7). Based on the above-reported data, the lactone-pretreated group was not included in this experiment.

### 2.6. Bioinformatic Evaluation of Possible Molecular Targets

Supplementary Tables S1 and S2 show that based on bioinformatic analysis there is no specific molecular target for BW-HS-101 and BW-iHS-101, respectively, with probability of interaction higher than 0.12 (calculated scores 0–0.11) [29].

## 3. Discussion

H_2_S as an endogenous gaseous mediator has been shown to be involved in regulation of many physiological functions within the cardiovascular and digestive systems [30,31,32]. H_2_S-releasing compounds were reported to exert anti-inflammatory and anti-oxidative properties under experimental conditions [33,34,35,36]. NaHS was shown to protect gastric mucosa against damage induced by ASA, alendronates, ethanol or by the exposure to stress or ischemia/reperfusion [37,38,39,40]. This chemical, together with Lawesson’s reagent, has been also reported to accelerate gastric ulcer healing [24]. Nevertheless, the balance between beneficial/toxic effects of H_2_S was strictly dose-dependent [22,41]. Therefore, over the last few years, several H_2_S-prodrugs were developed, capable of releasing this molecule in a somewhat controllable manner [27,42,43,44]. GYY4137, a pharmacological H_2_S donor, was observed to protect GI tract against acute oxidative injury and to exert chemoprevention effects [22,45]. A novel mitochondria-targeted H_2_S donor, AP-39, was shown to be protective against myocardial reperfusion injury [46]. Interestingly, new derivatives of NSAIDs were developed, such as H_2_S-releasing naproxen (ATB-346) or ketoprofen (ATB-352) [26,47]. These compounds were shown to have reduced GI toxicity with comparable or even more effective anti-inflammatory activity [26,47].

In our study, we have investigated for the first time the gastroprotective effect of novel organic H_2_S-prodrug, BW-HS-101 against the damage induced by the exposure to ASA or ethanol. BW-HS-101 is an esterase-sensitive compound releasing H_2_S in a controllable manner with a half-life of 13 min [27]. Importantly, esterases were reported to be active within gastric mucosa [48,49]. We observed that i.g. topical pretreatment with BW-HS-101 or BW-iHS-101 as its analogue without the ability to release H_2_S before exposure to a high dose of ASA (125 mg/kg i.g.) protected gastric mucosa against the damage induced by this NSAID. Moreover, gastroprotection of BW-HS-101 was further confirmed since this H_2_S-prodrug effectively prevented necrotic gastric mucosal damage induced by i.g. application of 75% ethanol. This effect of H_2_S-prodrug was accompanied by elevated GBF. Interestingly, BW-HS-101 did not reverse gastric mucosal PGE_2_ production, which is inhibited by well-known ASA activity. Pretreatment with BW-HS-101 decreased ASA-induced DNA oxidation in gastric mucosa. This is consistent with previously published data showing that NaHS decreased lipid peroxidation in the stomach induced by topical administration of ASA [50]. Our bioinformatic analysis of possible molecular targets of BW-HS-101 did not reveal any protein target that BW-HS-101 could interact with (calculated scores 0–0.11). Thus, we focused on oxidative- and inflammatory-response markers specific for gastric mucosal barrier maintenance, selected based on our previously published data [51]. BW-HS-101 exerted anti-inflammatory effects on the systemic level observed as decreased serum contents of IL-1β, TNF-α, IL-10, or VEGFA and locally as expressed by decreased gastric mucosal mRNA fold changes for IL-1R2 and COX-2. Importantly, BW-HS-101 maintained elevated anti-oxidative HMOX-1 and further enhanced anti-inflammatory SOCS3 mRNA expression increased in gastric mucosa exposed to ASA. Additionally, pretreatment with BW-HS-101 maintained upregulated mRNA expression of iNOS as a result of ASA administration. HMOX is an enzyme involved in the endogenous production of another gaseous mediator, CO [52]. While iNOS activity results in generation of endogenous NO [53]. Moreover, CO and NO, together with H_2_S were reported to contribute to the maintenance of gastric mucosal integrity and in modulation of gastric microcirculation [13]. Interestingly, our study revealed that pharmacological inhibition of NOS but not HMOX activity reversed the gastroprotective and vasodilatory effects of BW-HS-101. Thus, we assume that BW-HS-101 exerts gastroprotective activity via NO/NOS and SOCS3 pathways. However, CO/HMOX-1 contribution in BW-HS-101-mediated gastroprotection may exist, but not to a significant degree. Additionally, it has been reported previously that the H_2_S donor, NaHS, reduced chronic heart failure, possibly due to upregulation of HMOX-1 mRNA expression [54]. Moreover, NaHS-mediated gastroprotection against ASA-induced gastric damage has been shown to be NO biosynthesis-dependent [50].

Interestingly, we observed in our study that BW-iHS-101, chemically not able to release H_2_S, exerted similar gastroprotection against ASA-induced erosions on the microscopic level as compared with BW-HS-101. BW-iHS-101 also elevated gastric microcirculation but to a lower extent than BW-HS-101. Nevertheless, both compounds did not affect gastric mucosal PGE_2_ production and both decreased mRNA expression and/or serum concentration of inflammatory response markers such as IL-1R2, IL-1β, TNF-α, IL-10, and VEGFA, in parallel with reduced DNA oxidation. The bioinformatic analysis performed for BW-iHS-101 did not indicate any possible target protein (calculated scores 0–0.11). As it has been reported previously, BW-HS-101 or BW-iHS-101 is converted in the presence of esterase to a lactone derivative and H_2_S or H_2_O, respectively (Figure 1) [27]. Therefore, to evaluate the potential activity related with the biological effects of the tested compounds, we also evaluated the lactone for its possible molecular effectiveness within the gastric mucosa. We have found that the lactone derivative was biologically inactive. Our data has shown that pretreatment with lactone itself did not reduce the gastric damage score and did not affect GBF. Moreover, this compound was not effective in terms of inflammatory response inhibition since IL-1β, TNF-α, IL-10, VEGFA serum concentration and IL-R1, TNF-R2, IL-1R1, IL-1R2, and iNOS gastric mucosal mRNA expression were not decreased after subsequent administration of ASA. Importantly, in parallel with decreased NSAID-induced gastrotoxicity, BW-HS-101 and BW-iHS-101 maintained upregulated gastric mucosal mRNA expression for anti-oxidative HMOX-1 as well as increased concentration of EGF protein. However, H_2_S-releasing BW-HS-101 but not BW-iHS-101 upregulated gastric mucosal mRNA expression of anti-inflammatory SOCS-3 accompanied by decreased gastric mucosal concentration of inflammatory markers such as IL-1α, IL-1β, IL-2, IL-4, IL-6, IL-10, GM-CSF, IFNγ, TNF-α. This suggests that due to its ability to release H_2_S, BW-HS-101 is an effective gastroprotective compound with a different anti-inflammatory molecular pattern compared to BW-iHS-101.

According to experimental protocol, there was about 1.5–2 h between the pretreatments and the termination of experiments. Over this relatively short period of time, we were unable to observe any possible side effects of BW-HS-101 or BW-iHS-101 administration and toxicological analysis was not within the scope of our study.

## 4. Conclusions

**Figure 8:**
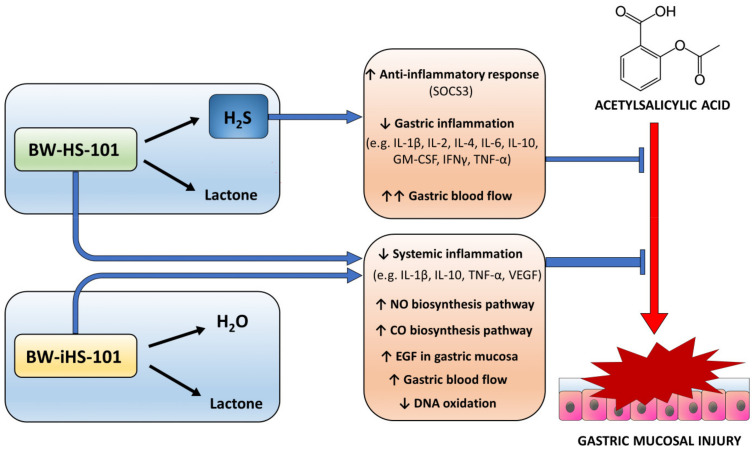
Overview on the possible mechanisms of BW-HS-101 and BW-iHS-101 gastroprotection against acetylsalicylic acid-induced injury. Abbreviations: H_2_S—hydrogen sulfide, NO—nitric oxide, CO—carbon monoxide, SOCS3—suppressor of cytokine signaling 3, IL—interleukin, TNF—tumor necrosis factor, VEGF—vascular endothelial growth factor A, EGF—epidermal growth factor, GM-CSF—granulocyte-macrophage colony-stimulating factor.

We conclude that possibly because of the similar chemical structure both, BW-HS-101 and BW-iHS-101 exert gastroprotective effect against NSAID-induced gastric damage. Nevertheless, due to the ability of BW-HS-101 to release H_2_S, this compound, in contrast to BW-iHS-101, more effectively upregulated gastric microcirculation and induced anti-oxidative and anti-inflammatory pathways as reflected by increased gastric mucosal mRNA expression for HMOX-1 and SOCS3, respectively, possibly leading to inhibition of local inflammatory response within gastric mucosa exposed to ASA (Figure 8).

## 5. Materials and Methods

### 5.1. Experimental Design

Experimental design involved fifty-five male Wistar rats with the age of 8–10 weeks and with average weight of 250–300 g. According to experimental protocol there was approximately 1.5–2 h between the first treatment and the termination of the experiment. Thus, weight changes were not assumed to be significantly altered after such a short period of time. Animals were fasted for 24 h with free access to tap water before each experiment. All procedures were approved by the 1st Local Ethical Committee for Care and Use of Experimental Animals, held by Faculty of Pharmacy, Jagiellonian University Medical College in Cracow (Decision No.: 311/2019; Date: 17 July 2019). Experiments were run with implications for replacement, refinement, or reduction (the 3Rs) principle and in compliance with the ARRIVE guidelines. Rats were randomly assigned to the appropriate experimental groups (five rats each) and were pretreated i.g. by orogastric tube with (1) 1mL of dimethyl sulfoxide (DMSO)/H_2_O (1:9) as vehicle, (2–4) H_2_S prodrug BW-HS-101 (0.5–50 μmol/kg), (5) its analogue, BW-iHS-101, without the ability to release H_2_S or (6) inactive metabolite of these compounds (lactone). BW-iHS-101 and lactone were applied at a dose of 50 μmol/kg, which is the equivalent of the effective dose of BW-HS-101 capable of reducing aspirin-induced gastric injury area by more than 50%. BW-HS-101 (50 μmol/kg) was also administered i.g. in combination with HMOX inhibitor, (7) ZnPP (10 mg/kg i.p.), or NOS inhibitor, (8) L-NNA (20 mg/kg i.p.), applied with the dose regimen based on previously described experiments [50]. After 30 min, animals were administered i.g. with 1.5 mL of ASA (125 mg/kg dissolved in 0.2 M HCl), based on previously implemented and described experimental model of ASA-induced gastric mucosal damage [50]. In separate experimental series, rats were pretreated i.g. with (9) vehicle or (10) BW-HS-101 (50 μmol/kg) 30 min before i.g. administration of 1 mL of 75% ethanol [28]. Because the molecular effects of BW-HS-101 and BW-iHS-101 on HMOX-1 and iNOS mRNA expression were similar, additional experimental groups with BW-iHS-101 + L-NNA or BW-iHS-101 + ZnPP were not employed to also comply with the 3R principle (by reduced number of animals involved in this protocol). Additionally, rats administered i.g. with vehicle without any additional treatments or gastric injury were considered as (11) the intact group.

All compounds and chemicals were purchased from Sigma Aldrich (Schnelldorf, Germany) unless otherwise stated. BW-HS-101 and BW-iHS-101 were synthesized by the Wang group (Georgia State University, Atlanta, GA, USA) following procedures described previously [27].

### 5.2. BW-HS-101 and BW-iHS-101 Synthesis and Chemical Conversion to Lactone

BW-HS-101 and BW-iHS-101 were synthesized following procedures described previously [27].

Conversion of BW-iHS-101 into the lactone was performed in vitro. Briefly, into the solution of a BW-iHS-101 (0.26 mmol) in MeOH (1.3 mL), 2 N NaOH (1.3 mL) was added, and the reaction was stirred for 2 h. Next, 10% HCl was added to acidify the reaction solution to the pH < 2. This acidified solution with precipitated crystals was extracted with ethyl acetate (13 mL × 3). The combined organic layers were washed with water (13 mL), dried (Na_2_SO_4_), filtered, and concentrated to yield a white solid product, which was characterized by LC-QTOF-MS analysis, using a 1200 series chromatograph and 6520 accurate-Mass QTOFMS (Agilent Technologies, Santa Clara, CA, USA), with the identification of the [M + H^+^] peak (205.1028), corresponding to the calculated mass of protonated lactone. In the LC chromatogram, the lactone was the only product with a retention time of 6.2 min.

### 5.3. GBF Determination, Microscopic Gastric Damage Assessment and Biological Samples Collection

One hour after administration of ASA or ethanol, under isoflurane anesthesia, the abdomen was opened for the GBF measurement by laser flowmetry, as described previously [55]. Briefly, the GBF was determined in the oxyntic part of the gastric mucosa using laser flowmeter (Laserflo, model BPM2, Blood Perfusion Monitor, Vasamedics, Saint Paul, MN, USA). Average values of three measurements were expressed as % of the average value determined in healthy gastric mucosa (% of control). Serum samples were collected from the vena cava and was stored at −80 °C until further analysis [55]. Stomach was excised, opened along the greater curvature and gastric mucosal samples were scraped off on ice, snap-frozen in liquid nitrogen and stored at −80 °C until further analysis [50]. For microscopic analysis, the gastric tissue sections were excised and fixed in 10% buffered formalin, pH 7.4. Samples were dehydrated by passing them through a series of alcohols with incremental concentrations, equilibrated in xylene for 10–15 min and embedded in paraffin; paraffin blocks were cut into about 4 μm sections using a microtome. The prepared specimens were stained with haematoxylin/eosin (H&E). Tissue slides were evaluated using a light microscope (AxioVert A1, Carl Zeiss, Oberkochen, Germany) [56]. Digital documentation of histological slides was obtained using ZEN Pro 2.3 software (Carl Zeiss, Oberkochen, Germany) [56].

All erosions/necrotic or inflammatory spots were evaluated based on following scoring criteria:

0 no erosion/necrosis/inflammation
1 length of injury <250 μm
2 length of injury 251–500 μm
3 length of injury 501–2000 μm

and

0 no erosion/necrosis/inflammation
1 depth of injury <500 μm per tissue section
2 depth of injury >500 μm per slide
3 depth of injury—erosion reaching submucosal layer

Median for the sum of abovementioned scoring criteria for all injuries in each slide separately was taken for further data analysis.

### 5.4. Determination of Gastric Mucosal mRNA Fold Changes by Real-Time PCR

Gastric mucosal mRNA expression fold changes for iNOS, HMOX-1, SOCS3, IL1-R1, IL1-R2, TNF-R2, COX-1, and COX-2 were assessed by real time PCR, as described previously [56]. Briefly, total RNA was isolated using commercially available kit with spin-columns (GeneMATRIX Universal RNA Purification Kit, EURx, Gdansk, Poland) according to manufacturer’s protocols. Reversed transcription (RT) was performed using PrimeScript™RTMasterMix (Perfect Real Time Takara Bio Inc., Kyoto, Japan). RNA concentration was measured using Qubit 4 Fluorometer (Thermo Fisher Scientific, Waltham, MA, USA). For each RT reaction, total RNA concentration was adjusted to (1 μg) per sample. Samples from healthy (intact) gastric mucosa were further used as reference control during calculations. Expression of mRNA for iNOS, HMOX-1, SOCS3, IL1-R1, IL1-R2, TNF-R2, COX-1, COX-2 and succinate dehydrogenase complex, subunit A (SDHA) and β-actin (ACTB) as reference genes was determined using specific primers as described previously [50,51,57]. PCR reaction was run using thermal cycler Quant Studio 3 (Thermo Fisher Scientific, Waltham, MA, USA) and SYBR Green I dye including kit (SG qPCR Master Mix (2×), EURx, Gdansk, Poland). To maintain the same PCR reaction efficiency in all analyzed samples, the same amount of cDNA per each well was used. After reaction, melting curve for each sample, its technical replicates and for appropriate negative control were analyzed to exclude the data derived from potentially unintended products. Results were analyzed using the −∆∆Ct method [58].

### 5.5. Luminex Microbeads Fluorescent Assays

Determination of serum concentrations of interleukin (IL)-1β, IL10, tumor necrosis factor (TNF)-α and vascular endothelial growth factor A (VEGF) was performed using Luminex microbeads fluorescent assay (Bio-Rad, Hercules, CA, USA) and Luminex MAGPIX system (Luminex Corp., Austin, TX, USA). Results were calculated from calibration curves and expressed in pg/mL [28]. Gastric mucosal concentrations of IL-1α, IL-1β, IL-2, IL-4, IL-6, IL-10, IL-13, GM-CSF, IFN, TNF-α, EGF, and VEGF were determined with the implementation of calibration curve. All samples were standardized in terms of total protein concentration before the assay and results were expressed as pg/mL of the tissue homogenate [28].

### 5.6. Determination of PGE_2_ and 8-OHdG Concentration in Gastric Mucosa

PGE_2_ and 8-OdHG concentrations in gastric mucosa were determined as described in detail previously [51]. Briefly, PGE_2_ concentrations in gastric mucosal samples obtained from the ulcer margin were determined using PGE_2_ ELISA kit (ab133021, Abcam, Cambridge, UK) according to the manufacturer’s protocol. The homogenization process of each sample was standardized regarding sample weight and buffer volume and results were expressed in pg/mL of gastric tissue homogenate. 8-OHdG content as DNA oxidation marker was assessed in DNA isolated from gastric mucosa using ELISA kit (589320, Cayman Chemical, Ann Arbor, MI, USA) according to manufacturer’s protocol.

### 5.7. Bioinformatic Analysis of Possible Molecular Targets of BW-HS-101

Possible molecular targets of BW-HS-101 were evaluated using SwissTargetPrediction, which estimates the probability for a tested compound to have indicated protein as a molecular target. Calculated scores higher than 0.5 indicate that the investigated compounds are likely to interact with given protein [59].

### 5.8. Statistical Analysis

Experiments and data collection were done by operators blinded to the sample identity. Results were analyzed using GraphPad Prism 5.0 software (GraphPad Software Inc., La Jolla, CA, USA). Results are presented as mean ± SEM and as median ± range (Figure 2). Statistical analysis was conducted using Student’s *t*-test or ANOVA with Dunnett’s multiple comparison or Bonferroni post hoc tests if more than two experimental groups were compared. Kruskal–Wallis test was used for the data shown on Figure 2. The size for each experimental group was of *n* = 5. *p* < 0.05 was considered as statistically significant.

## Figures and Tables

**Table 1:** Gastric blood flow (GBF) in gastric mucosa of rats pretreated i.g. with vehicle, BW-HS-101 (50 μmol/kg), BW-iHS-101 (50 μmol/kg), lactone (50 μmol/kg), or BW-HS-101 applied in combination with nitroarginine (L-NNA, 20 mg/kg i.p.) or zinc protoporphyrin IX (ZnPP, 10 mg/kg i.p.), 30 min before i.g. administration of acetylsalicylic acid (ASA, 125 mg/kg). Intact refers to the values obtained in healthy gastric mucosa without ASA-induced gastric damage. Results are mean ± SEM of five rats per group. Significant changes as compared with the respective values in intact gastric mucosa are indicated by asterisk (*) (*p* < 0.05). Hash (^#^) indicates significant changes as compared with vehicle administered with ASA (*p* < 0.05). Hat (^) indicates significant difference as compared with BW-HS-101 (*p* < 0.05).

Experimental Group	GBF [% of Control]
Intact	99.99 ± 2.371
Vehicle + ASA	58.45 ± 2.795 *
BW-HS-101 + ASA	81.16 ± 1.232 ^#^
BW-iHS-101 + ASA	70.05 ± 2.646 ^#^^
Lactone + ASA	64.25 ± 1.963
BW-HS-101 + L-NNA + ASA	66.18 ± 3.206 ^
BW-HS-101 + ZnPP + ASA	84.54 ± 4.113

## Data Availability

All the data supporting the conclusions is included within the manuscript and is available on request from the corresponding author.

## Supplementary Materials

The following are available online at https://www.mdpi.com/article/10.3390/ijms22105211/s1, Table S1: Target prediction data for BW-HS-101, Table S2: Target prediction data for BW-iHS-101.
