# Supplementary material for: Novel Hydrogen Sulfide (H2S)-Releasing BW-HS-101 and Its Non-H2S Releasing Derivative in Modulation of Microscopic and Molecular Parameters of Gastric Mucosal Barrier
Source: Int J Mol Sci. 2021 May 14;22(10):5211. doi: 10.3390/ijms22105211 (PMC8155842; doi:10.3390/ijms22105211)
Supplement: Supplementary file 1 [file ijms-22-05211-s001.zip › Table S2_SwissTargetPrediction-BW-iHS-101.pdf]

Target Prediction data for BW-iHS-101

SwissTargetPrediction

Target	Common name	Uniprot ID	ChEMBL ID	Target Class	Probability*	Known actives (3D/2D)
Protein tyrosine kinase 2 beta	PTK2B	Q14289	CHEMBL5469	Kinase	0.100578902067	2 / 0
Aldehyde dehydrogenase	ALDH2	P05091	CHEMBL1935	Oxidoreductase	0.100578902067	0 / 1
c-Jun N-terminal kinase 3	MAPK10	P53779	CHEMBL2637	Kinase	0.100578902067	12 / 0
Cytochrome P450 11B2	CYP11B2	P19099	CHEMBL2722	Cytochrome P450	0.100578902067	23 / 0
G-protein coupled bile acid receptor 1	GPBAR1	Q8TDU6	CHEMBL5409	Family A G protein-coupled receptor	0.100578902067	15 / 0
Glutaminyl-peptide cyclotransferase-like protein	QPCTL	Q9NXS2	CHEMBL3638349	Transferase	0.100578902067	6 / 0
Serotonin 5a (5-HT5a) receptor	HTR5A	P47898	CHEMBL3426	Family A G protein-coupled receptor	0.100578902067	23 / 0
Butyrylcholinesterase	BCHE	P06276	CHEMBL1914	Hydrolase	0.100578902067	16 / 0
Poly [ADP-ribose] polymerase-1	PARP1	P09874	CHEMBL3105	Enzyme	0.100578902067	37 / 0
Kinesin-like protein 1	KIF11	P52732	CHEMBL4581	Other cytosolic protein	0.100578902067	17 / 0
Hepatocyte growth factor receptor	MET	P08581	CHEMBL3717	Kinase	0.100578902067	11 / 0
Metabotropic glutamate receptor 1	GRM1	Q13255	CHEMBL3772	Family C G protein-coupled receptor	0.100578902067	4 / 0
Isocitrate dehydrogenase [NADP] cytoplasmic	IDH1	O75874	CHEMBL2007625	Enzyme	0.100578902067	4 / 0
Tyrosine-protein kinase JAK3	JAK3	P52333	CHEMBL2148	Kinase	0.100578902067	106 / 0
NAD-dependent deacetylase sirtuin 2	SIRT2	Q8IXJ6	CHEMBL4462	Eraser	0.100578902067	7 / 18
Nischarin	NISCH	Q9Y2I1	CHEMBL3923	Other cytosolic protein	0.100578902067	48 / 0
Glutamate NMDA receptor; GRIN1/ GRIN2B	GRIN1 GRIN2B	Q05586 Q13224	CHEMBL1907603	Ligand-gated ion channel	0.100578902067	13 / 0
Alpha-2b adrenergic receptor	ADRA2B	P18089	CHEMBL1942	Family A G protein-coupled receptor	0.100578902067	84 / 0
Epoxide hydratase	EPHX2	P34913	CHEMBL2409	Protease	0.100578902067	17 / 0
Intercellular adhesion molecule-1	ICAM1	P05362	CHEMBL3070	Adhesion	0.100578902067	2 / 0
Selectin E	SELE	P16581	CHEMBL3890	Adhesion	0.100578902067	2 / 0
Thymidylate synthase	TYMS	P04818	CHEMBL1952	Transferase	0.100578902067	50 / 0
Beta-secretase 1	BACE1	P56817	CHEMBL4822	Protease	0.100578902067	109 / 0
Toll-like receptor 8	TLR8	Q9NR97	CHEMBL5805	Toll-like and Il-1 receptors	0.100578902067	1 / 0
Focal adhesion kinase 1	PTK2	Q05397	CHEMBL2695	Kinase	0.100578902067	8 / 0
Delta opioid receptor	OPRD1	P41143	CHEMBL236	Family A G protein-	0.100578902067	24 / 0

Target	Common name	Uniprot ID	ChEMBL ID	Target Class	Probability*	Known actives (3D/2D)
				coupled receptor		
Mu opioid receptor	OPRM1	P35372	CHEMBL233	Family A G protein-coupled receptor	0.100578902067	18 / 0
Adenosine A2b receptor	ADORA2B	P29275	CHEMBL255	Family A G protein-coupled receptor	0.100578902067	3 / 0
Vascular endothelial growth factor receptor 2	KDR	P35968	CHEMBL279	Kinase	0.100578902067	45 / 0
Cyclin-dependent kinase 2	CDK2	P24941	CHEMBL301	Kinase	0.100578902067	4 / 0
Cyclin-dependent kinase 1	CDK1	P06493	CHEMBL308	Kinase	0.100578902067	12 / 0
Voltage-gated potassium channel subunit Kv1.5	KCNA5	P22460	CHEMBL4306	Voltage-gated ion channel	0.100578902067	7 / 0
Alpha-1d adrenergic receptor	ADRA1D	P25100	CHEMBL223	Family A G protein-coupled receptor	0.0	36 / 0
Alpha-1b adrenergic receptor	ADRA1B	P35368	CHEMBL232	Family A G protein-coupled receptor	0.0	34 / 0
Serine/threonine-protein kinase 17B	STK17B	O94768	CHEMBL3980	Kinase	0.0	12 / 0
Estradiol 17-beta-dehydrogenase 3	HSD17B3	P37058	CHEMBL4234	Enzyme	0.0	12 / 0
Serine/threonine-protein kinase 17A	STK17A	Q9UEE5	CHEMBL4525	Kinase	0.0	12 / 0
Trace amine-associated receptor 1 (by homology)	TAAR1	Q96RJ0	CHEMBL5857	Family A G protein-coupled receptor	0.0	94 / 0
Phospholipase A2 group IIA	PLA2G2A	P14555	CHEMBL3474	Enzyme	0.0	11 / 0
C-X-C chemokine receptor type 3	CXCR3	P49682	CHEMBL4441	Family A G protein-coupled receptor	0.0	25 / 0
Glutaminyl-peptide cyclotransferase	QPCT	Q16769	CHEMBL4508	Enzyme	0.0	51 / 0
Muscarinic acetylcholine receptor M5	CHRM5	P08912	CHEMBL2035	Family A G protein-coupled receptor	0.0	3 / 0
Dopamine D1 receptor	DRD1	P21728	CHEMBL2056	Family A G protein-coupled receptor	0.0	16 / 0
Muscarinic acetylcholine receptor M2	CHRM2	P08172	CHEMBL211	Family A G protein-coupled receptor	0.0	3 / 0
Muscarinic acetylcholine receptor M1	CHRM1	P11229	CHEMBL216	Family A G protein-coupled receptor	0.0	7 / 0
Serine/threonine-protein kinase Aurora-B	AURKB	Q96GD4	CHEMBL2185	Kinase	0.0	7 / 0
Tyrosine-protein kinase SRC	SRC	P12931	CHEMBL267	Kinase	0.0	15 / 0
ALK tyrosine kinase receptor	ALK	Q9UM73	CHEMBL4247	Kinase	0.0	6 / 0
Serine/threonine-	NEK6	Q9HC98	CHEMBL4309	Kinase	0.0	1 / 0

Target	Common name	Uniprot ID	ChEMBL ID	Target Class	Probability*	Known actives (3D/2D)
protein kinase NEK6						
Uridine phosphorylase 1 (by homology)	UPP1	Q16831	CHEMBL4811	Enzyme	0.0	12 / 0
Sodium/hydrogen exchanger 1	SLC9A1	P19634	CHEMBL2781	Electrochemical transporter	0.0	14 / 0
Serotonin 1d (5-HT1d) receptor	HTR1D	P28221	CHEMBL1983	Family A G protein-coupled receptor	0.0	18 / 0
Serotonin 7 (5-HT7) receptor	HTR7	P34969	CHEMBL3155	Family A G protein-coupled receptor	0.0	25 / 0
Cytochrome P450 26A1	CYP26A1	O43174	CHEMBL5141	Cytochrome P450	0.0	2 / 0
Cyclin-dependent kinase 5/CDK5 activator 1	CDK5R1 CDK5	Q15078 Q00535	CHEMBL1907600	Kinase	0.0	20 / 0
Cyclin-dependent kinase 1/cyclin B	CCNB3 CDK1 CCNB1 CCNB2	Q8WWL7 P06493 P14635 O95067	CHEMBL2094127	Other cytosolic protein	0.0	14 / 0
Apoptosis regulator Bcl-X	BCL2L1	Q07817	CHEMBL4625	Other ion channel	0.0	1 / 0
Apoptosis regulator Bcl-2	BCL2	P10415	CHEMBL4860	Other ion channel	0.0	4 / 0
Prostanoid EP2 receptor	PTGER2	P43116	CHEMBL1881	Family A G protein-coupled receptor	0.0	0 / 17
Prostanoid EP3 receptor	PTGER3	P43115	CHEMBL3710	Family A G protein-coupled receptor	0.0	0 / 13
Estradiol 17-beta-dehydrogenase 2	HSD17B2	P37059	CHEMBL2789	Enzyme	0.0	27 / 0
Lysine-specific demethylase 4D-like	KDM4E	B2RXH2	CHEMBL1293226	Eraser	0.0	1 / 0
Kappa Opioid receptor	OPRK1	P41145	CHEMBL237	Family A G protein-coupled receptor	0.0	33 / 0
Cathepsin D	CTSD	P07339	CHEMBL2581	Protease	0.0	2 / 0
Melanocortin receptor 4	MC4R	P32245	CHEMBL259	Family A G protein-coupled receptor	0.0	2 / 0
Dual specificity protein phosphatase 3	DUSP3	P51452	CHEMBL2635	Phosphatase	0.0	6 / 0
Serine/threonine-protein kinase PAK 1	PAK1	Q13153	CHEMBL4600	Kinase	0.0	1 / 0
Serine/threonine-protein kinase Aurora-C	AURKC	Q9UQB9	CHEMBL3935	Kinase	0.0	6 / 0
Serine/threonine-protein kinase Aurora-A	AURKA	O14965	CHEMBL4722	Kinase	0.0	7 / 0
Carbonic anhydrase I	CA1	P00915	CHEMBL261	Lyase	0.0	87 / 0
G-protein coupled receptor 84	GPR84	Q9NQS5	CHEMBL3714079	Family A G protein-coupled receptor	0.0	19 / 0
Nitric oxide synthase, inducible	NOS2	P35228	CHEMBL4481	Enzyme	0.0	28 / 0

Target	Common name	Uniprot ID	ChEMBL ID	Target Class	Probability*	Known actives (3D/2D)
Vanilloid receptor	TRPV1	Q8NER1	CHEMBL4794	Voltage-gated ion channel	0.0	39 / 0
Muscarinic acetylcholine receptor M4	CHRM4	P08173	CHEMBL1821	Family A G protein-coupled receptor	0.0	2 / 0
Tyrosine-protein kinase JAK2	JAK2	O60674	CHEMBL2971	Kinase	0.0	81 / 0
Cytochrome P450 2C19	CYP2C19	P33261	CHEMBL3622	Cytochrome P450	0.0	2 / 0
Calcium-activated potassium channel subunit alpha-1	KCNMA1	Q12791	CHEMBL4304	Voltage-gated ion channel	0.0	11 / 0
Voltage-gated potassium channel subunit Kv1.3	KCNA3	P22001	CHEMBL4633	Voltage-gated ion channel	0.0	7 / 0
Glycogen synthase kinase-3 beta	GSK3B	P49841	CHEMBL262	Kinase	0.0	11 / 0
Nitric-oxide synthase, brain	NOS1	P29475	CHEMBL3568	Enzyme	0.0	29 / 0
Nitric-oxide synthase, endothelial	NOS3	P29474	CHEMBL4803	Enzyme	0.0	11 / 0
Aldehyde dehydrogenase 1A1 (by homology)	ALDH1A1	P00352	CHEMBL3577	Enzyme	0.0	1 / 0
Dihydropyrimidine dehydrogenase	DPYD	Q12882	CHEMBL3172	Enzyme	0.0	1 / 0
Carbonic anhydrase VI	CA6	P23280	CHEMBL3025	Lyase	0.0	3 / 0
Phosphodiesterase 10A (by homology)	PDE10A	Q9Y233	CHEMBL4409	Phosphodiesterase	0.0	13 / 0
Serotonin 2a (5-HT2a) receptor (by homology)	HTR2A	P28223	CHEMBL224	Family A G protein-coupled receptor	0.0	31 / 25
Receptor protein-tyrosine kinase erbB-2	ERBB2	P04626	CHEMBL1824	Kinase	0.0	14 / 0
Epidermal growth factor receptor erbB1	EGFR	P00533	CHEMBL203	Kinase	0.0	46 / 0
5-lipoxygenase activating protein	ALOX5AP	P20292	CHEMBL4550	Other cytosolic protein	0.0	10 / 0
GABA-A receptor; alpha-5/beta-3/gamma-2	GABRB3 GABRG2 GABRA5	P28472 P18507 P31644	CHEMBL2094122	Ligand-gated ion channel	0.0	5 / 0
Fibroblast growth factor receptor 1	FGFR1	P11362	CHEMBL3650	Kinase	0.0	9 / 0
PH domain leucine-rich repeat-containing protein phosphatase 2	PHLPP2	Q6ZVD8	CHEMBL1275209	Enzyme	0.0	1 / 0
Macrophage migration inhibitory factor	MIF	P14174	CHEMBL2085	Enzyme	0.0	5 / 0
Serine-protein kinase	ATR	Q13535	CHEMBL5024	Kinase	0.0	3 / 0

Target	Common name	Uniprot ID	ChEMBL ID	Target Class	Probability*	Known actives (3D/2D)
ATR						
Peroxisome proliferator-activated receptor gamma	PPARG	P37231	CHEMBL235	Nuclear receptor	0.0	0 / 100
Peroxisome proliferator-activated receptor delta	PPARD	Q03181	CHEMBL3979	Nuclear receptor	0.0	0 / 27
Tubulin beta-1 chain	TUBB1	Q9H4B7	CHEMBL1915	Structural protein	0.0	3 / 0
Histamine H3 receptor	HRH3	Q9Y5N1	CHEMBL264	Family A G protein-coupled receptor	0.0	57 / 0
Histamine H4 receptor	HRH4	Q9H3N8	CHEMBL3759	Family A G protein-coupled receptor	0.0	43 / 0
3-phosphoinositide dependent protein kinase-1	PDPK1	O15530	CHEMBL2534	Kinase	0.0	4 / 0
