# Supplementary material for: Novel Hydrogen Sulfide (H2S)-Releasing BW-HS-101 and Its Non-H2S Releasing Derivative in Modulation of Microscopic and Molecular Parameters of Gastric Mucosal Barrier
Source: Int J Mol Sci. 2021 May 14;22(10):5211. doi: 10.3390/ijms22105211 (PMC8155842; doi:10.3390/ijms22105211)
Supplement: Supplementary file 1 [file ijms-22-05211-s001.zip › Table S1_SwissTargetPrediction -BW-HS-101.pdf]

Target Prediction data for BW-HS-101

SwissTargetPrediction

Target	Common name	Uniprot ID	ChEMBL ID	Target Class	Probability*	Known actives (3D/2D)
Vanilloid receptor	TRPV1	Q8NER1	CHEMBL4794	Voltage-gated ion channel	0.100578902067	342 / 0
Rho-associated protein kinase 2	ROCK2	O75116	CHEMBL2973	Kinase	0.100578902067	80 / 0
Muscarinic acetylcholine receptor M1 (by homology)	CHRM1	P11229	CHEMBL216	Family A G protein-coupled receptor	0.100578902067	116 / 0
Leucine-rich repeat serine/threonine-protein kinase 2	LRRK2	Q5S007	CHEMBL1075104	Kinase	0.100578902067	78 / 0
Gamma-secretase	PSEN2<br>PSENEN<br>NCSTN<br>APH1A<br>PSEN1<br>APH1B	P49810<br>Q9NZ42<br>Q92542<br>Q96BI3<br>P49768<br>Q8WW43	CHEMBL2094135	Protease	0.100578902067	227 / 0
Rho-associated protein kinase 1	ROCK1	Q13464	CHEMBL3231	Kinase	0.100578902067	74 / 0
Epoxide hydratase	EPHX2	P34913	CHEMBL2409	Protease	0.100578902067	278 / 0
Trace amine-associated receptor 1	TAAR1	Q96RJ0	CHEMBL5857	Family A G protein-coupled receptor	0.100578902067	109 / 0
Protein kinase C gamma (by homology)	PRKCG	P05129	CHEMBL2938	Kinase	0.100578902067	54 / 0
Bromodomain-containing protein 4	BRD4	O60885	CHEMBL1163125	Reader	0.100578902067	106 / 0
Tyrosine-protein kinase ITK/TSK	ITK	Q08881	CHEMBL2959	Kinase	0.100578902067	42 / 0
Translocator protein (by homology)	TSPO	P30536	CHEMBL5742	Membrane receptor	0.100578902067	477 / 0
Protein kinase C beta	PRKCB	P05771	CHEMBL3045	Kinase	0.100578902067	16 / 0
Serine/threonine-protein kinase Aurora-B	AURKB	Q96GD4	CHEMBL2185	Kinase	0.100578902067	64 / 0
Kinesin-like protein 1	KIF11	P52732	CHEMBL4581	Other cytosolic protein	0.100578902067	65 / 0
Serine/threonine-protein kinase Aurora-A	AURKA	O14965	CHEMBL4722	Kinase	0.100578902067	95 / 0
Sodium channel protein type V alpha subunit	SCN5A	Q14524	CHEMBL1980	Voltage-gated ion channel	0.100578902067	23 / 0
Sodium channel protein type II alpha subunit	SCN2A	Q99250	CHEMBL4187	Voltage-gated ion channel	0.100578902067	30 / 0
Cytochrome P450 11B2	CYP11B2	P19099	CHEMBL2722	Cytochrome P450	0.100578902067	311 / 0
Adenosine A1 receptor (by	ADORA1	P30542	CHEMBL226	Family A G protein-coupled receptor	0.100578902067	504 / 0

Target	Common name	Uniprot ID	ChEMBL ID	Target Class	Probability*	Known actives (3D/2D)
homology)						
Neurokinin 1 receptor	TACR1	P25103	CHEMBL249	Family A G protein-coupled receptor	0.100578902067	68 / 0
Melanocortin receptor 4	MC4R	P32245	CHEMBL259	Family A G protein-coupled receptor	0.100578902067	24 / 0
Poly [ADP-ribose] polymerase-1	PARP1	P09874	CHEMBL3105	Enzyme	0.100578902067	280 / 0
TGF-beta receptor type I	TGFBR1	P36897	CHEMBL4439	Kinase	0.100578902067	115 / 0
Signal transducer and activator of transcription 3	STAT3	P40763	CHEMBL4026	Transcription factor	0.100578902067	55 / 0
Alkaline phosphatase, tissue-nonspecific isozyme	ALPL	P05186	CHEMBL5979	Enzyme	0.100578902067	44 / 0
Vascular endothelial growth factor receptor 2	KDR	P35968	CHEMBL279	Kinase	0.100578902067	482 / 0
MAP kinase ERK2	MAPK1	P28482	CHEMBL4040	Kinase	0.100578902067	56 / 0
Potassium channel subfamily K member 3	KCNK3	O14649	CHEMBL2321613	Voltage-gated ion channel	0.100578902067	37 / 0
Potassium channel subfamily K member 9	KCNK9	Q9NPC2	CHEMBL2321614	Voltage-gated ion channel	0.100578902067	28 / 0
MAP kinase p38 alpha	MAPK14	Q16539	CHEMBL260	Kinase	0.100578902067	531 / 0
Mixed lineage kinase 7	MAP3K20	Q9NYL2	CHEMBL3886	Kinase	0.100578902067	5 / 0
Tyrosine-protein kinase ABL2	ABL2	P42684	CHEMBL4014	Kinase	0.100578902067	2 / 0
TGF-beta receptor type II	TGFBR2	P37173	CHEMBL4267	Kinase	0.100578902067	5 / 0
Carnitine O-palmitoyltransferase 1, liver isoform	CPT1A	P50416	CHEMBL1293194	Enzyme	0.100578902067	71 / 0
Cholecystokinin B receptor (by homology)	CCKBR	P32239	CHEMBL298	Family A G protein-coupled receptor	0.100578902067	195 / 0
Phosphodiesterase 10A	PDE10A	Q9Y233	CHEMBL4409	Phosphodiesterase	0.100578902067	700 / 0
Phosphodiesterase 4B	PDE4B	Q07343	CHEMBL275	Phosphodiesterase	0.100578902067	90 / 0
Phosphodiesterase 7A	PDE7A	Q13946	CHEMBL3012	Phosphodiesterase	0.100578902067	80 / 0
Heat shock protein HSP 90-alpha	HSP90AA1	P07900	CHEMBL3880	Other cytosolic protein	0.100578902067	64 / 0
Neuropeptide Y receptor type 5	NPY5R	Q15761	CHEMBL4561	Family A G protein-coupled receptor	0.100578902067	261 / 0
Endothelin-converting enzyme 2	ECE2	O60344	CHEMBL5890	Protease	0.100578902067	2 / 0
Cyclophilin A (by homology)	PPIA	P62937	CHEMBL1949	Isomerase	0.100578902067	50 / 0

Target	Common name	Uniprot ID	ChEMBL ID	Target Class	Probability*	Known actives (3D/2D)
Tyrosine-protein kinase JAK3	JAK3	P52333	CHEMBL2148	Kinase	0.100578902067	326 / 0
Tyrosine-protein kinase JAK1	JAK1	P23458	CHEMBL2835	Kinase	0.100578902067	167 / 0
Tyrosine-protein kinase JAK2	JAK2	O60674	CHEMBL2971	Kinase	0.100578902067	343 / 0
JAK3/JAK1	JAK3 JAK1	P52333 P23458	CHEMBL3038491	Kinase	0.100578902067	20 / 0
Cytochrome P450 19A1	CYP19A1	P11511	CHEMBL1978	Cytochrome P450	0.100578902067	640 / 0
Serine/threonine-protein kinase PIM1	PIM1	P11309	CHEMBL2147	Kinase	0.100578902067	159 / 0
c-Jun N-terminal kinase 1	MAPK8	P45983	CHEMBL2276	Kinase	0.100578902067	209 / 0
c-Jun N-terminal kinase 3	MAPK10	P53779	CHEMBL2637	Kinase	0.100578902067	165 / 0
Histamine H3 receptor	HRH3	Q9Y5N1	CHEMBL264	Family A G protein-coupled receptor	0.100578902067	95 / 0
Histamine H4 receptor	HRH4	Q9H3N8	CHEMBL3759	Family A G protein-coupled receptor	0.100578902067	83 / 0
c-Jun N-terminal kinase 2	MAPK9	P45984	CHEMBL4179	Kinase	0.100578902067	104 / 0
11-beta-hydroxysteroid dehydrogenase 1	HSD11B1	P28845	CHEMBL4235	Enzyme	0.100578902067	585 / 0
Serine/threonine-protein kinase PIM2	PIM2	Q9P1W9	CHEMBL4523	Kinase	0.100578902067	98 / 0
Neprilysin (by homology)	MME	P08473	CHEMBL1944	Protease	0.100578902067	11 / 0
Androgen Receptor	AR	P10275	CHEMBL1871	Nuclear receptor	0.100578902067	171 / 0
Cyclin-dependent kinase 5/CDK5 activator 1	CDK5R1 CDK5	Q15078 Q00535	CHEMBL1907600	Kinase	0.100578902067	178 / 0
Cyclin-dependent kinase 4/cyclin D1	CCND1 CDK4	P24385 P11802	CHEMBL1907601	Kinase	0.100578902067	63 / 0
GABA-A receptor; alpha-5/beta-3/gamma-2	GABRB3 GABRG2 GABRA5	P28472 P18507 P31644	CHEMBL2094122	Ligand-gated ion channel	0.100578902067	147 / 0
Cathepsin K	CTSK	P43235	CHEMBL268	Protease	0.100578902067	293 / 0
CaM kinase II	CAMK2D	Q13557	CHEMBL2801	Kinase	0.100578902067	6 / 0
Protein kinase C alpha	PRKCA	P17252	CHEMBL299	Kinase	0.100578902067	43 / 0
Cyclin-dependent kinase 1	CDK1	P06493	CHEMBL308	Kinase	0.100578902067	70 / 0
Protein kinase C epsilon	PRKCE	Q02156	CHEMBL3582	Kinase	0.100578902067	4 / 0
MAP kinase-interacting serine/threonine-protein kinase MNK1	MKNK1	Q9BUB5	CHEMBL4718	Kinase	0.100578902067	15 / 0
Mineralocorticoid receptor	NR3C2	P08235	CHEMBL1994	Nuclear receptor	0.100578902067	120 / 0

Target	Common name	Uniprot ID	ChEMBL ID	Target Class	Probability*	Known actives (3D/2D)
Progesterone receptor	PGR	P06401	CHEMBL208	Nuclear receptor	0.100578902067	246 / 0
Hormone sensitive lipase	LIPE	Q05469	CHEMBL3590	Enzyme	0.100578902067	58 / 0
Fibroblast growth factor receptor 1	FGFR1	P11362	CHEMBL3650	Kinase	0.100578902067	64 / 0
Estradiol 17-beta-dehydrogenase 3	HSD17B3	P37058	CHEMBL4234	Enzyme	0.100578902067	41 / 0
Tyrosine-protein kinase BRK	PTK6	Q13882	CHEMBL4601	Kinase	0.100578902067	20 / 0
Caspase-3	CASP3	P42574	CHEMBL2334	Protease	0.100578902067	85 / 0
Nitric-oxide synthase, brain	NOS1	P29475	CHEMBL3568	Enzyme	0.100578902067	85 / 0
Quinone reductase 2	NQO2	P16083	CHEMBL3959	Enzyme	0.100578902067	56 / 0
Nitric oxide synthase, inducible	NOS2	P35228	CHEMBL4481	Enzyme	0.100578902067	129 / 0
Nitric-oxide synthase, endothelial	NOS3	P29474	CHEMBL4803	Enzyme	0.100578902067	27 / 0
Cytochrome P450 11B1	CYP11B1	P15538	CHEMBL1908	Cytochrome P450	0.100578902067	248 / 0
Adenosine A2a receptor	ADORA2A	P29274	CHEMBL251	Family A G protein-coupled receptor	0.100578902067	355 / 0
Cytochrome P450 24A1 (by homology)	CYP24A1	Q07973	CHEMBL4521	Enzyme	0.100578902067	4 / 0
Serotonin 2b (5-HT2b) receptor	HTR2B	P41595	CHEMBL1833	Family A G protein-coupled receptor	0.100578902067	104 / 0
GABA receptor alpha-1 subunit (by homology)	GABRA1	P14867	CHEMBL1962	Ligand-gated ion channel	0.100578902067	31 / 0
Cyclin-dependent kinase 1/cyclin B	CCNB3 CDK1 CCNB1 CCNB2	Q8WWL7 P06493 P14635 O95067	CHEMBL2094127	Other cytosolic protein	0.100578902067	117 / 0
PI3-kinase p110-alpha/p85-alpha	PIK3CA PIK3R1	P42336 P27986	CHEMBL2111367	Enzyme	0.100578902067	11 / 0
Muscarinic acetylcholine receptor M2 (by homology)	CHRM2	P08172	CHEMBL211	Family A G protein-coupled receptor	0.100578902067	50 / 0
Serotonin 2c (5-HT2c) receptor	HTR2C	P28335	CHEMBL225	Family A G protein-coupled receptor	0.100578902067	169 / 0
Glycogen synthase kinase-3 beta	GSK3B	P49841	CHEMBL262	Kinase	0.100578902067	302 / 0
PI3-kinase p110-alpha subunit	PIK3CA	P42336	CHEMBL4005	Enzyme	0.100578902067	158 / 0
GABA receptor alpha-5 subunit (by homology)	GABRA5	P31644	CHEMBL5112	Ligand-gated ion channel	0.100578902067	24 / 0
Toll-like receptor (TLR7/TLR9)	TLR9	Q9NR96	CHEMBL5804	Toll-like and Il-1 receptors	0.100578902067	32 / 0
Pyruvate kinase	PKM	P14618	CHEMBL1075189	Enzyme	0.100578902067	36 / 0

Target	Common name	Uniprot ID	ChEMBL ID	Target Class	Probability*	Known actives (3D/2D)
isozymes M1/M2						
Serine/threonine-protein kinase RAF	RAF1	P04049	CHEMBL1906	Kinase	0.100578902067	50 / 0
Beta-secretase 1	BACE1	P56817	CHEMBL4822	Protease	0.100578902067	311 / 0
Muscarinic acetylcholine receptor M4	CHRM4	P08173	CHEMBL1821	Family A G protein-coupled receptor	0.100578902067	18 / 0
Vascular endothelial growth factor receptor 1	FLT1	P17948	CHEMBL1868	Kinase	0.100578902067	122 / 0
Muscarinic acetylcholine receptor M5	CHRM5	P08912	CHEMBL2035	Family A G protein-coupled receptor	0.100578902067	16 / 0
Carbonic anhydrase II	CA2	P00918	CHEMBL205	Lyase	0.100578902067	467 / 0
Cyclin-dependent kinase 2/cyclin E	CCNE2 CDK2 CCNE1	O96020 P24941 P24864	CHEMBL2094126	Other cytosolic protein	0.100578902067	76 / 0
Dual-specificity tyrosine-phosphorylation regulated kinase 1A	DYRK1A	Q13627	CHEMBL2292	Kinase	0.100578902067	103 / 0
